# The power of artificial intelligence for managing pandemics: A primer for public health professionals

**DOI:** 10.1002/hpm.3864

**Published:** 2024-10-27

**Authors:** Martin McKee, Rikard Rosenbacke, David Stuckler

**Affiliations:** ^1^ European Observatory on Health Systems and Policies London School of Hygiene and Tropical Medicine London UK; ^2^ Department of Accounting Centre for Corporate Governance Copenhagen Business School Frederiksberg Denmark; ^3^ Department of Social and Political Science Bocconi University Milano Italy

**Keywords:** artificial intelligence, healthcare management, pandemic, predictive modelling, public health

## Abstract

Artificial intelligence (AI) applications are complex and rapidly evolving, and thus often poorly understood, but have potentially profound implications for public health. We offer a primer for public health professionals that explains some of the key concepts involved and examines how these applications might be used in the response to a future pandemic. They include early outbreak detection, predictive modelling, healthcare management, risk communication, and health surveillance. Artificial intelligence applications, especially predictive algorithms, have the ability to anticipate outbreaks by integrating diverse datasets such as social media, meteorological data, and mobile phone movement data. Artificial intelligence‐powered tools can also optimise healthcare delivery by managing the allocation of resources and reducing healthcare workers' exposure to risks. In resource distribution, they can anticipate demand and optimise logistics, while AI‐driven robots can minimise physical contact in healthcare settings. Artificial intelligence also shows promise in supporting public health decision‐making by simulating the social and economic impacts of different policy interventions. These simulations help policymakers evaluate complex scenarios such as lockdowns and resource allocation. Additionally, it can enhance public health messaging, with AI‐generated health communications shown to be more effective than human‐generated messages in some cases. However, there are risks, such as privacy concerns, biases in models, and the potential for ‘false confirmations’, where AI reinforces incorrect decisions. Despite these challenges, we argue that AI will become increasingly important in public health crises, but only if integrated thoughtfully into existing systems and processes.

## Highlights

- Artificial intelligence (AI) can help with early detection of outbreaks with pandemic potential
- Predictive AI can enhance healthcare logistics and resource use
- AI can simulate policies and inform public health decisions
- AI‐powered messages can improve health communication

## 1 INTRODUCTION

A recent editorial in this journal called for more analysis of the applications and implications of artificial intelligence (AI) in policy, planning and management.^1^ In this paper we respond to that call by exploring the use of AI in one particular context, the public health response to a pandemic. The COVID‐19 pandemic brought home the importance of data and the ability to analyse and interpret it. Major decisions, such as imposing restrictions on movement, were made in virtual real‐time using data from many sources to track the spread of infection, movement of people, public perceptions, and much else. The quantity of data could easily become overwhelming.

Our starting point is that AI will inevitably play a greater role in the public health response to any future crisis, whether this takes the form of a pandemic or something else, such as a natural disaster or a major conflict. When the pandemic began in 2020, AI was still playing a limited role, but it has advanced rapidly. However, this poses challenges for those in leadership roles in public health. It is a highly technical area where knowledge and its applications are rapidly changing. It is also one where there is an exceptionally large information asymmetry between those responsible for procuring the technology and those who produce it.

These characteristics offer both opportunities and risks. The opportunities which we describe in this review are considerable and, we contend, cannot be ignored, especially given the evidence that all countries could have done better in the pandemic and the imperative to be better prepared for future crises. The risks are that they invest limited resources, both financial and human, in technologies that, at best, do not work and, at worst, do harm by increasing risks of cyberattacks or data breaches or giving rise to incorrect and potentially dangerous recommendations.

In this review, we explore briefly some of its possible roles. These include early detection and predictive modelling of outbreaks, optimising healthcare delivery and management, public health decision support, health surveillance, and risk communications and messaging.

This is a vast and highly technical field, so all that is possible in a brief review is to provide a basic introduction and review what is possible. Our expectation is that it will provide an agenda for more detailed discussions by those in leadership roles in public health about how AI can be systematically integrated into public health infrastructures for pandemic management. By exploring AI's role in prediction, resource allocation, and outbreak tracking, we build on the growing literature on AI's transformative potential in decision‐making processes while balancing the risk of excessive trust in AI‐generated advice.^2^ In these ways, we outline how AI can support policymakers, managers, and public health professionals, facilitating more informed decisions during health crises.

## 2 THE TYPES OF AI AND WAYS OF LEARNING

AI encompasses techniques for performing tasks that typically require human intelligence, such as recognising speech and images, making decisions, and translating languages. It has been classified in various ways. One of the most parsimonious has three groups: Artificial Narrow Intelligence, Artificial General Intelligence, and Artificial Superintelligence.^3^

Artificial Narrow Intelligence (sometimes referred to as Weak AI) performs a discrete task or a set of closely related tasks. It cannot generalise its skills beyond its specific domain. Examples include voice assistants, like Siri or Alexa, which use natural language processing to respond to user queries; recommendation systems, such as algorithms used by Netflix or Spotify to suggest things that reflect user preferences; and image recognition systems that can identify objects, faces, or scenes, and autonomous vehicles, that combine sensors, machine learning, and decision‐making algorithms. It also includes Large Language Model (LLMs)s, which learn languages by looking for statistical associations within texts. Most current applications of AI fall within this category. They can perform well on standard tests. Thus Roivainen asked ChatGPT to take an IQ test and it achieved a very respectable score of 155.^4^ However, it was unable to answer the question ‘who is the father of Sebastian's children?’, which required reasoning.

Artificial General Intelligence (sometimes referred to as Strong AI) was initially conceptualised as a means to replicate human cognitive abilities that could understand, learn, and apply knowledge across many tasks, much like a human, and engage in reasoning, problem‐solving, and abstract thinking.^5^ However, this has been supplanted by a recognition that biological and AI are fundamentally different. For example, machines are unable to apply intuition, moral judgement (unless pre‐programed to do so), and self‐awareness, but they can process large amounts of data much faster than humans. It is also unclear how it would be determined whether a machine possesses Artificial General Intelligence, with proposed tests ranging from the well‐known Turing Test, where those conversing with it cannot distinguish whether it is a machine or a human, to the Coffee Test, where a machine can enter a house, find what is needed, and make a coffee.

There is also what is sometimes referred to as Artificial Superintelligence. This represents a hypothetical future where machines surpass human intelligence in creativity, problem‐solving, and emotional intelligence. Although this has attracted much attention, it is questionable whether it can ever be achieved. Thus, for now, the main applications to pandemic management will be powered through predictive AI algorithms similar to those currently employed by Netflix and Apple.

It is also important to note how AI systems are generated to better understand their applications. These AI systems are powered by sophisticated algorithms that enable them to ‘train’ on large datasets to learn rapidly. These include four main types of learning (Box 1).

**Box 1 Types of learning used in artificial intelligence**

1 *Machine learning* is an application of AI that allows computers to extract knowledge from data autonomously.^6^ Machine learning algorithms analyse ‘training data’ to make predictions or decisions without being explicitly programed for the task.^7^ They use three main methods: supervised learning, unsupervised learning, and reinforcement learning.

*Supervised learning*, the most common, uses algorithms trained on a labelled dataset, with each training example paired with an output label. The goal is to predict the labels of new, unseen data. The most common uses involve regression (linear, logistic, or polynomial) to predict things like house prices based on features like size, location, and age, or classification, used to allocate things to categories, for example, to classify emails as spam or not spam. Supervised learning works best when there is a large amount of labelled data and the relationship between the input and output is well understood, such as detection of common anomalies on medical images.

*Unsupervised learning* deals with unlabelled data to infer a structure present in a set of data points. One example is clustering, which groups data points based on their similarity, for example, to segment customers into distinct groups to target marketing material. A second is dimensionality, which reduces the number of variables being considered. These can be used, for example, to identify clusters of patients with similar characteristics in electronic health records.^8^ A third is anomaly detection, which identifies rare items, events, or observations that raise suspicions by differing significantly from the majority of the data. These can be used, for example, to identify possible fraudulent data or system failures or, in the present context, to identify breakdowns in wastewater treatment where problems are easily concealed by noise in the system.^9^ Unsupervised learning is particularly useful when dealing with large datasets where the goal is to explore and understand the data's structure in the absence of predefined labels.

*Reinforcement learning* is where an agent learns to make decisions by performing certain actions and receiving rewards or penalties. The objective is to learn a policy that maximises the cumulative reward over time. Value‐Based Methods focus on estimating the value of actions, to find the best action to take given the current state. Policy‐based methods optimise the policy that the agent follows. These work best when the scope for action is large. Model‐based methods involve building a model of the environment and using it for planning. Reinforcement learning is widely used in the gaming industry, but applications in health are beginning to emerge, illustrated by the development of a model that incorporates physiological data and clinical expertise to diagnose sepsis.^10^

We now look at some areas in which these applications could be used in a future pandemic.

## 3 APPLICATION 1: EARLY DETECTION AND PREDICTIVE MODELLING OF OUTBREAKS

Quite possibly the first digital surveillance of epidemics took place in 1994, when ProMED (Programme for Monitoring Emerging Infectious Diseases), a network‐based tool, was created to identify unusual health events related to emerging and re‐emerging infectious diseases.^11^ It was developed following recognition that the earliest signals of an emerging health crisis often appeared in reports from the media, professional networks, and health professionals. Thus, it drew upon crowdsourced, global reporting, building on an email list, field reports, and website. On 30 December 2019 ProMED reported chatter on the Chinese microblogging site, Weibo, about cases of pneumonia in Wuhan whose cause was unknown.^12^ Thus it was among the first to report on COVID‐19 before it was officially recognised as a virus with pandemic potential. Although ProMED is still manually curated, it illustrates how the growth of social media has vastly expanded the material that can be analysed. The tool epitweetr, developed by the European Centre for Disease Prevention and Control (ECDC) to detect public health threats from X (formerly Twitter) is an example.^13^

Historically, infectious disease surveillance was handicapped by an unwillingness by some governments to report outbreaks. These are now much more difficult to conceal. Similar developments have taken place in other sectors, including conflict, law enforcement, and economic forecasting, using Open Source Intelligence (OSINT).^14^ This has enabled non‐governmental organisations, such as Bellingcat, to provide rapid assessments of events in conflict zones previously restricted to state intelligence agencies,^15^ more important than ever given the growth of disinformation.^16^ However, the enormous expansion of data sources also poses problems for analysis. This is an obvious candidate for the use of AI, now being taken forward by a growing number of platforms.^17^

The potential of AI goes beyond the detection of outbreaks. By combining data on topics such as weather, vector distribution, movement of individuals, and living conditions, times and places where there is a greater risk of an outbreak can be identified. This could always be done, for example, using the link between malaria and altitude or rainy seasons, but greater computing power, sophisticated algorithms, and new data sources have transformed it.^18^ These include tracking people's movement using signals from their mobile phones,^19^ satellite imagery offering near real‐time data on physical infrastructure and land use, and detailed meteorological data.^20^

Looking beyond COVID‐19, a recent study from Southern Nigeria identified precipitation, elevation, population density, and temperature variations as environmental conditions suitable for mPox with high predictive accuracy.^21^ However, even without incorporating such environmental variables, a machine learning algorithm was shown to outperform traditional time‐series methods in predicting the course of a mPox outbreak.^22^ de la Lastra and colleagues have reviewed the wide range of potential applications of AI in tackling antimicrobial resistance, including identifying pathogens, understanding resistance patterns, predicting treatment outcomes, and discovering new antibiotics.^23^ Machine learning algorithms can also analyse genomic data to identify genetic markers associated with antimicrobial resistance, aiding in development of targeted treatment strategies.

Yet, as Syrowatka and colleagues note in a 2021 scoping review, AI has certain limitations, particularly in terms of data integration, system scalability, and ensuring data quality.^24^ AI‐driven prediction models depend on the availability of appropriate high‐quality data from sources such as medical records, disease notifications, and laboratory results. However, these datasets vary widely in structure and quality, posing significant challenges in training AI models. For instance, many COVID‐19 models reviewed were found to be based on data from specific settings (e.g., hospital admissions) and should not have been generalised, resulting in high risks of bias and overfitting.^25^ Furthermore, there is a lack of standardisation in how data is collected across different healthcare systems, which complicates the validation and application of these models beyond the initial training environment. A particular concern is the absence, from many datasets, of variables capturing ethnicity, given the known risk that AI perpetuates hidden bias.^26^ Another major issue is missing data, commonly handled inappropriately by performing complete‐case analyses that further reduce model reliability.^25^ Addressing these challenges requires improving data quality, ensuring the availability of diverse datasets, and adopting robust validation strategies to enhance model performance and applicability across different healthcare settings.

## 4 APPLICATION 2: OPTIMISING HEALTHCARE DELIVERY AND MANAGEMENT

Distribution of materials, such as medicines and personal protection equipment, was challenging during the pandemic. First, scarce resources needed to be allocated optimally. Second, transport and logistics workers' exposure to risks of infection needed to be minimised. AI can, to some extent, address both.

### 4.1 Managing scarce resources to meet evolving demand

AI can be used in predictive models that anticipate changes in demand and adjust distribution patterns accordingly. During the pandemic, researchers working with a large retail chain used this approach to detect panic‐buying behaviours using data from the company, such as online orders and promotions, and external data, such as online searches and social media posts.^27^ They were able to adjust distribution of essential products (e.g. toilet paper, canned soup, and household cleaners) and increase access substantially.

While this example sought to optimise the performance of an established logistic system, the challenges are much greater in an emergency where there is severe damage to infrastructure and people are displaced. Zahedi and colleagues developed a decision‐making model that simultaneously addressed goods distribution and vehicle routing and validated it using data from the 2017 Kermanshah earthquake in Iran.^28^ An obvious challenge in such circumstances is the lack of historical data to train the algorithms, but Huang and Song have described how they incorporated expert judgements on demand and travel times into their model designed to optimise the distribution of essential goods.^29^

A public health emergency will increase demand for health facilities. Those responsible for allocating scarce resources must simultaneously match the differing needs of many patients for oxygen, isolation, or monitoring to available resources in a rapidly changing situation. Although the use of AI in this area is still at an early stage, proof of concept has been demonstrated in virtual simulations.^30^ A related application is the use of AI to analyse patient characteristics in ways that identify hospitalised patients expected to have prolonged length of stay, pointing to those who may need earlier interventions^31^, ^32^ or who are showing the first signs of deteriorating.^33^ While considerable caution is required, AI may also be able to help in a situation where health workers are scarce. LLMs power chatbots, which have been rated higher than physician responses in terms of quality and empathy in some limited circumstances.^34^ However, at best their use will be limited to the simplest of tasks.^35^

### 4.2 Safer distribution of medicine and protective equipment

Every encounter between a patient and a health or care worker in a pandemic presents an opportunity to transmit infection. This has encouraged research on greater use of robots,^36^ a process that has been gathering pace in recent years in Japan, a country with a rapidly ageing population that faces a severe workforce shortage.^37^ This can take many forms, from measures that ease the activities of daily living, taking advantage of the Internet of Things, now used widely in the automation of homes, to chatbots that can provide advice and action requests,^38^ and to physical robots that can undertake cleaning and disinfection, deliver medicines and shopping, and assist with mobility.^39^ However, caution is required, as evidence suggests that many of the initial claims about robots' contributions to personal care have not been realised. For example, robots designed to move residents increased staff workload, and those intended to engage with and stimulate them were abandoned as they became boring.^40^

### 4.3 Reducing demands on health workers

The pandemic has had profound consequences for frontline health workers who often toiled for long hours in hot and uncomfortable personal protective equipment, experiencing high levels of burnout.^41^ AI, taking advantage of advances in areas such as voice recognition, offers potential to automate many routine clinical tasks, such as notetaking.

## 5 APPLICATION 3: PUBLIC HEALTH DECISION SUPPORT

Human behaviour in an emergency is intrinsically complex, with patterns determined by starting conditions (path dependency), non‐linear changes, variable lags between interventions and outcomes, and positive and negative feedback loops. People make decisions in ways that reflect their perceptions and those of people around them, the scale and nature of threats, and constraints to different courses of action. For example, while politicians debated when to impose movement restrictions at the onset of the COVID‐19 pandemic, many people acted before they were instructed to.^42^

Such restrictions involve trade‐offs. While interrupting transmission of a highly infectious microorganism is essential, the associated restrictions have consequences for health, disproportionately affecting those already disadvantaged.^43^

Agent‐based social simulations model the interactions of autonomous agents to create virtual societies. These can help evaluate the potential consequences of different policy interventions.^44^ Dignum and colleagues report on how such a tool can explore trade‐offs involved in the closure of schools or work‐from‐home orders.^45^

Similarly, Song and colleagues explored how city‐wide or community lockdowns and restrictions on types of movement could limit disease transmission while minimising economic damage.^46^ It created an AI oracle using reinforcement learning to identify solutions that were then modelled under different assumptions relating to the complexity of implementation, impact on health services, and costs in relation to benefits.

## 6 APPLICATION 4: HEALTH SURVEILLANCE

Although few countries captured data on social and economic characteristics of their populations, it was clear that the pandemic was shining a light on long‐standing inequalities, with worse outcomes among those leading precarious lives. However, many of the analyses that have been undertaken have focused on individual characteristics, such as income, employment status, or ethnicity. Disadvantage is characterised by intersectionality, in which characteristics, at both the individual and the community level, interact.

Bowser and colleagues used machine learning to cluster over 650 variables from 24 different databases, capturing geographic data in the United States.^47^ They identified three broad clusters, with markedly different life expectancies. These differed from those identified previously by investigator‐led analyses. Importantly, they showed that health system infrastructure was an important contributor to the cluster with the highest life expectancy, a finding with policy relevance as the United States grapples with hospital closures in many areas.

In the United States, ensemble machine learning has also been used to aggregate county‐level data on physical and mental health, environmental pollution, access to health care, demographic characteristics, and other epidemiological data to explore correlates of COVID‐19 outcomes. As would be expected, given pervasive structural racism, the proportion of African Americans was a strong predictor of adverse outcomes, but less intuitively, so was greater use of public transport.^48^ In Germany, Doblhammer and colleagues used a similar approach to show how those at risk of adverse COVID‐19 outcomes differed at successive stages of the pandemic.^49^

## 7 APPLICATION 5: IMPROVED PUBLIC HEALTH RISK COMMUNICATIONS AND MESSAGING

AI can enhance the quality, clarity, and effectiveness of health‐related messages. LLMs have been employed to generate health awareness messages that are on par with or even surpass human‐generated content. For example, AI‐generated messages about folic acid were found to be superior in message quality and clarity than the most retweeted human‐generated messages.^50^ AI has also been used to create persuasive COVID‐19 vaccination messages, perceived as more effective and evoking more positive attitudes than those authored by human institutions like the US Centres for Disease Control.^51^ AI can also be used to analyse data from social media to track sentiments and beliefs^52^ and identify misinformation.^53^ Finally, AI has been used to develop virtual simulated patients, providing a cost‐effective and resource‐efficient platform to practice and enhance communication skills.^54^

## 8 OTHER APPLICATIONS

The examples examined above cannot be exhaustive. There are many applications of AI in widespread use that offer scope to simplify tasks during a pandemic, just as they do in normal times. These include searching for literature, preparing documents, creating images to convey messages and so on. However, many of these applications are underused and a 2020 survey found that 68% of data in commercial enterprises remains unused.^55^ The figure in many health systems is likely to be higher. Processing electronic health records is particularly challenging because it contains structured and unstructured text, images, and laboratory data.^56^

There are also many basic science and clinical applications, although these go beyond the scope of this paper with its focus on the public health response. Many of these have been described in detail in a recent review by Lv and colleagues.^57^ To take a few examples, the, OpenSAFELY platform categorised patients into 10 groups based on the extent and nature of antibiotic use in the 3 years prior to a COVID‐19 diagnosis and found an almost fivefold difference in the probability of severe COVID‐19 in those at each end of the scale.^58^ Machine learning is also being advocated for use in the discovery of putative drugs.^59^ This offers a means to predict properties of compounds such as binding affinity and toxicity without needing to synthesise them.^60^ However, while several novel molecules are now in the advanced stages of evaluation, AI so far has been, at best, an adjunct to the conventional human‐led approach.^61^ The use of AI in drug discovery also poses challenges for regulatory bodies relating to standards for data quality, transparency, and model validation, all of which take time that is limited in a crisis.^62^ Consequently, the most promising application of AI in a pandemic may be screening existing drugs to identify priorities for subsequent biological testing.^63^

## 9 DREAM OR REALITY?

So far, we have explored the potential opportunities that AI may offer in a public health emergency. However, this does not mean that this potential will be realised.

While some of the methods that we have described are now in widespread use, such as the algorithms used to make recommendations in online shopping, these are situations where the stakes are low. This is not the case in a public health emergency where an incorrect decision can cost many lives. Moreover, these uses benefit from a vast amount of data collected in real‐time from customers, often linked to large volumes of other information on them, such as their posts on social media. In contrast, an emergency such as a pandemic is, to a considerable extent, uncharted territory.

It is also important to recognise that many of the applications discussed above have not been comprehensively evaluated or validated. Often, they are at best proof of concept. Van Smeden and colleagues have usefully provided a list of 12 questions that anyone evaluating AI‐based prediction models should ask. These relate to conceptualisation, data collection, predictors and outcomes, openness and fairness, reporting, and model performance.^64^ Looking ahead, this task may be eased by adoption of standardised reporting systems, such as TRIPOD + AI (Transparent Reporting of a multivariable prediction model for Individual Prognosis Or Diagnosis).^65^ However, given the pace of change, it is important that policymakers know what to look for.

It is also necessary to be aware of the many risks associated with AI. The processing of vast quantities of often sensitive personal information poses obvious risks to data privacy and security. This can lead to identity theft, extortion, and other serious consequences for those whose data is disclosed. Like any electronic resource, systems using AI are at risk of cyber attacks or software failures, such as those arising from the software update in July 2024 that brought down computer systems worldwide.^66^

AI also poses risks to equity, especially where models are trained on data that is itself biased. Thus, there are many examples of diagnostic algorithms trained on datasets that exclude certain ethnic groups that generate erroneous responses when applied to patients in those groups.^67^ In some cases, this can perpetuate existing biases in treatment.

False confirmation errors occur when a decision‐maker (e.g., physicians or policymakers) makes an incorrect decision that is confirmed by faulty AI advice.^68^ These errors are a previously undiscovered form of human bias, akin to confirmation bias,^69^, ^70^ that results in uncritical acceptance of decisions without further scrutiny.^68^ While much attention has been focused on whether AI systems are correct or incorrect, especially when conflicting with evidence‐based medicine, it is essential to understand the implications of human‐AI collaboration fully.

Awareness of the risk of false confirmations is crucial for effective policy, planning, and management. It is often insidious in that it can reinforce confidence in potentially erroneous decisions. These errors appear to have a high prevalence, estimated to affect 5%–30% of decisions.^68^ Lopes and colleagues note that AI can assist insurance companies in assessing demographics, medical history, and behavioural factors but also that this is an area especially susceptible to false confirmation, potentially exacerbating inequality.^1^

Special attention is needed with LLMs, which can potentially both exacerbate and mitigate confirmation bias.^56^, ^71^, ^72^, ^73^ Ke and colleagues found that LLMs significantly reduced biases and improved diagnostic accuracy in simulated clinical decision‐making.^72^ However, there are risks like ‘hallucination’ when LLMs generate false content and when LLMS use research that is false, outdated and/or biased.^56^ Misleading LLMs can rapidly spread voluminous misinformation, targeting specific groups or even individuals.^74^ The introduction of LLMs in collaborative human‐AI decision‐making must be carefully evaluated,^72^ given that humans tend to favour compelling narratives, especially stories that align with our own views.^69^

AI systems will only be used if they are trusted sufficiently, but not so much that users fail to challenge them when responses appear wrong.^68^ This has given rise to the concept of explainable AI, in which the algorithms set out why they reached the decision or what might have made them reach a different one.^75^ However, at present, many AI systems function as black boxes, and the application of this concept remains at an early stage.

If AI is to be used in the ways set out, it must be integrated into existing structures and processes. Otherwise, there is a risk of workflow disruptions, inefficiencies, and additional demands on those operating the systems or applying the resulting decisions. Unfortunately, the history of failures in information technology procurement is not encouraging.^76^

Finally, most AI systems depend on the availability of raw data on the phenomenon they are addressing. Yet, what happens when the data has itself been generated by AI? This is a particular concern with generative AI, which produces images and text by scraping the Internet. Errors then accumulate with each iteration with AI‐generated data acting, in effect, as a poison.^77^

## 10 IMPLICATIONS FOR POLICY

We have reviewed the transformative role that AI can play in public health, especially in managing pandemics. It can help with early outbreak detection, resource allocation, healthcare delivery, and public health communication. However, for this to be achieved, policymakers must integrate AI into public health systems. Moreover, with these opportunities come significant responsibilities. Policymakers must develop robust frameworks to address privacy concerns related to the massive amounts of data AI requires, such as personal health records and real‐time social media monitoring. Data governance policies must ensure privacy, security, and ethical usage while minimising risks like cyberattacks and breaches. This issue is covered in detail in a report by the World Health Organisation.^78^

They must also be aware of the risks of bias in AI algorithms, which can lead to unequal healthcare outcomes if datasets used to train AI models do not represent diverse populations. Policies should mandate transparency in AI training methods and the use of diverse datasets to ensure equitable healthcare for all.

Another important consideration is the ethical collaboration between humans and AI in decision‐making. AI can reinforce incorrect decisions if not properly monitored, a phenomenon known as ‘false confirmation’. Therefore, policymakers must establish guidelines for the responsible use of AI, ensuring that decisions are validated and not blindly accepted from AI outputs. Trust in AI will be crucial for their effective adoption, especially in healthcare. Explainable AI, where algorithms can clarify how decisions are reached, should be prioritised in policy frameworks to build confidence among users, particularly in high‐stakes scenarios like pandemics.

AI has the potential to reduce strain on healthcare workers, automating routine tasks like documentation and optimising the distribution of critical resources. This has obvious attractions for policymakers. However, while they must explore how AI can be used to support overburdened health systems, especially during emergencies, they need to be cautious about unintended consequences of workforce disruption.

Ultimately, there is a need for forward‐thinking policies that integrate AI into pandemic preparedness strategies. By doing so, policymakers can leverage AI's ability to predict outbreaks, manage resources, and enhance public health messaging, while safeguarding against the risks inherent in its use.

## 11 CONCLUSION

AI will have profound consequences for public health. However, in such a rapidly evolving field, it is very difficult to separate the spin from the substance. And while it offers many opportunities, it also brings risks. The one thing about which we can be certain is that health policymakers of the future will have to acquire an understanding of this complex area. Evidence of how many of them struggled with even relatively straightforward mathematical principles and uncertainties during the pandemic, the challenges should not be underestimated.

## CONFLICT OF INTEREST STATEMENT

None.

## ETHICS STATEMENT

No human subject research was undertaken so ethics approval is not required.

## Data Availability

Data sharing not applicable to this article as no datasets were generated or analysed during the current study.

## References

[1] Lopes MA , Martins H , Correia T . Artificial intelligence and the future in health policy, planning and management. Int J Health Plann Manag. 2024;39(1):3‐8. 10.1002/hpm.3709 37749780

[2] Rosenbacke R , Melhus Å , McKee M , Stuckler D . How Explainable AI can increase or decrease clinicians’ trust in AI applications in healthcare: a systematic review. JMIR AI 2024. in press. 10.2196/53207 PMC11561425 39476365

[3] Girasa R . AI as a disruptive technology. In: Girasa R , ed. Artificial Intelligence as a Disruptive Technology: Economic Transformation and Government Regulation. Palgrave Macmillan; 2020:3‐21.

[4] Roivainen E . AI's IQ: ChatGPT aced a test but showed that intelligence cannot be measured by IQ alone. Sci Am. 2023;329(1):7. 10.1038/scientificamerican0723-7 39038127

[5] Russell SJ , Norvig P . Artificial Intelligence: A Modern Approach. Pearson; 2016.

[6] IBM . Machine learning versus deep learning versus neural networks. 2024. Accessed July 26, 2024. https://www.ibm.com/topics/machine‐learning

[7] Sarker IH . Machine learning: algorithms, real‐world applications and research directions. SN Computer Science. 2021;2(3):160. 10.1007/s42979-021-00592-x 33778771 PMC7983091

[8] Pavon JM , Previll L , Woo M , et al. Machine learning functional impairment classification with electronic health record data. J Am Geriatr Soc. 2023;71(9):2822‐2833. 10.1111/jgs.18383 37195174 PMC10524844

[9] Bellamoli F , Di Iorio M , Vian M , Melgani F . Machine learning methods for anomaly classification in wastewater treatment plants. J Environ Manag. 2023;344:118594. 10.1016/j.jenvman.2023.118594 37473555

[10] Wu X , Li R , He Z , Yu T , Cheng C . A value‐based deep reinforcement learning model with human expertise in optimal treatment of sepsis. NPJ Digit Med. 2023;6(1):15. 10.1038/s41746-023-00755-5 36732666 PMC9894526

[11] Carrion M , Madoff LC . ProMED‐mail: 22 years of digital surveillance of emerging infectious diseases. Int Health. 2017;9(3):177‐183. 10.1093/inthealth/ihx014 28582558 PMC5881259

[12] Zheng P , Adams PC , Wang J . Shifting moods on sina Weibo: the first 12 weeks of COVID‐19 in wuhan. New Media Soc. 2024;26(1):346‐367. 10.1177/14614448211058850

[13] Espinosa L , Wijermans A , Orchard F , et al. Epitweetr: early warning of public health threats using Twitter data. Euro Surveill. 2022;27(39). 10.2807/1560-7917.es.2022.27.39.2200177 PMC9524055 36177867

[14] Browne TO , Abedin M , Chowdhury MJM . A systematic review on research utilising artificial intelligence for open source intelligence (OSINT) applications. Int J Inf Secur. 2024;23(4):1‐28. 10.1007/s10207-024-00868-2

[15] Karan K . Open‐Source journalism: innovations and ethical challenges. In: Dahiya S , Trehan K , eds. Handbook of Digital Journalism: Perspectives from South Asia. Springer Nature Singapore; 2024:385‐395.

[16] Wang Y , Bye J , Bales K , et al. Understanding and neutralising covid‐19 misinformation and disinformation. BMJ. 2022;379:e070331. 10.1136/bmj-2022-070331 36414251

[17] MacIntyre CR , Chen X , Kunasekaran M , et al. Artificial intelligence in public health: the potential of epidemic early warning systems. J Int Med Res. 2023;51(3):03000605231159335. 10.1177/03000605231159335 36967669 PMC10052500

[18] Zhang T , Rabhi F , Behnaz A , et al. Use of automated machine learning for an outbreak risk prediction tool. Inform Med Unlocked. 2022;34:101121. 10.1016/j.imu.2022.101121

[19] Szocska M , Pollner P , Schiszler I , et al. Countrywide population movement monitoring using mobile devices generated (big) data during the COVID‐19 crisis. Sci Rep. 2021;11(1):5943. 10.1038/s41598-021-81873-6 33723282 PMC7961025

[20] Teillet C , Devillers R , Tran A , et al. Exploring fine‐scale urban landscapes using satellite data to predict the distribution of Aedes mosquito breeding sites. Int J Health Geogr. 2024;23(1):18. 10.1186/s12942-024-00378-3 38972982 PMC11229250

[21] Arotolu TE , Afe AE , Wang H , et al. Spatial modeling and ecological suitability of monkeypox disease in Southern Nigeria. PLoS One. 2022;17(9):e0274325. 10.1371/journal.pone.0274325 36126054 PMC9488772

[22] Qureshi M , Khan S , Bantan RAR , et al. Modeling and forecasting monkeypox cases using stochastic models. J Clin Med. 2022;11(21):6555. 10.3390/jcm11216555 36362783 PMC9659136

[23] de la Lastra JMP , Wardell SJT , Pal T , de la Fuente‐Nunez C , Pletzer D . From data to decisions: leveraging artificial intelligence and machine learning in combating antimicrobial resistance ‐ a comprehensive review. J Med Syst. 2024;48(1):71. 10.1007/s10916-024-02089-5 39088151 PMC11294375

[24] Syrowatka A , Kuznetsova M , Alsubai A , et al. Leveraging artificial intelligence for pandemic preparedness and response: a scoping review to identify key use cases. NPJ digital medicine. 2021;4(1):96. 10.1038/s41746-021-00459-8 34112939 PMC8192906

[25] Wynants L , Van Calster B , Collins GS , et al. Prediction models for diagnosis and prognosis of covid‐19: systematic review and critical appraisal. BMJ. 2020;369:m1328. 10.1136/bmj.m1328 32265220 PMC7222643

[26] Bozorgmehr K , McKee M , Azzopardi‐Muscat N , et al. Integration of migrant and refugee data in health information systems in Europe: advancing evidence, policy and practice. Lancet Reg Health Eur. 2023;34:100744. 10.1016/j.lanepe.2023.100744 37927430 PMC10625017

[27] Adulyasak Y , Benomar O , Chaouachi A , Cohen MC , Khern‐Am‐Nuai W . Using AI to Detect Panic Buying and Improve Products Distribution amid Pandemic. AI and society; 2023:1‐30. 10.1007/s00146-023-01654-9 PMC10105357 37358947

[28] Zahedi A , Kargari M , Kashan AH . Multi‐objective decision‐making model for distribution planning of goods and routing of vehicles in emergency multi‐objective decision‐making model for distribution planning of goods and routing of vehicles in emergency. Int J Disaster Risk Reduc. 2020;48:101587. 10.1016/j.ijdrr.2020.101587

[29] Huang X , Song L . An emergency logistics distribution routing model for unexpected events. Ann Oper Res. 2018;269(1‐2):223‐239. 10.1007/s10479-016-2300-7

[30] Accelerated Capability Environment . Using AI to improve patient bed allocation decisions in hospital. 2023. Accessed July 24, 2024. https://www.gov.uk/government/case‐studies/using‐ai‐to‐improve‐patient‐bed‐allocation‐decisions‐in‐hospital

[31] Lai CH , Mok PK , Chau WW , Law SW . Application of machine learning models on predicting the length of hospital stay in fragility fracture patients. BMC Med Inf Decis Making. 2024;24(1):26. 10.1186/s12911-024-02417-2 PMC10826155 38291406

[32] Orooji A , Shanbehzadeh M , Mirbagheri E , Kazemi‐Arpanahi H . Comparing artificial neural network training algorithms to predict length of stay in hospitalized patients with COVID‐19. BMC Infect Dis. 2022;22(1):923. 10.1186/s12879-022-07921-2 36494613 PMC9733380

[33] Spear J , Ehrenfeld JM , Miller BJ . Applications of artificial intelligence in health care delivery. J Med Syst. 2023;47(1):121. 10.1007/s10916-023-02018-y 37975946 PMC10656306

[34] Ayers JW , Poliak A , Dredze M , et al. Comparing physician and artificial intelligence chatbot responses to patient questions posted to a public social media forum. JAMA Intern Med. 2023;183(6):589‐596. 10.1001/jamainternmed.2023.1838 37115527 PMC10148230

[35] Amin R , Darwin R , Dey BK , Dhama K , Bin Emran T . Examining the differences between how doctors and artificial intelligence chatbots handle patient symptoms. Int J Surg. 2023;109(10):2892‐2895. 37352516 10.1097/JS9.0000000000000565 PMC10583905

[36] Javaid M , Haleem A , Singh RP , Rab S , Suman R , Kumar L . Utilization of robotics for healthcare: a scoping review. Journal of Industrial Integration and Management. 2022:2250015. 10.1142/s2424862222500154

[37] Ravankar AA , Tafrishi SA , Luces JVS , Seto F , Hirata Y . Care: cooperation of ai robot enablers to create a vibrant society. IEEE Robot Autom Mag. 2022;30(1):8‐23.

[38] Jovanović M , Baez M , Casati F . Chatbots as conversational healthcare services. IEEE Internet Computing. 2020;25(3):44‐51. 10.1109/mic.2020.3037151

[39] Jovanovic K , Schwier A , Matheson E , et al. Digital innovation hubs in health‐care robotics fighting COVID‐19: novel support for patients and health‐care workers across Europe. IEEE Robot Autom Mag. 2021;28(1):40‐47. 10.1109/mra.2020.3044965

[40] Wright J . Inside Japan’s long experiment in automating elder care. 2023. Accessed October 12, 2024. https://www.technologyreview.com/2023/01/09/1065135/japan‐automating‐eldercare‐robots/

[41] Clarke R . Breathtaking: Inside the NHS in a Time of Pandemic. Little, Brown and Company; 2021.

[42] Vannoni M , McKee M , Semenza JC , Bonell C , Stuckler D . Using volunteered geographic information to assess mobility in the early phases of the COVID‐19 pandemic: a cross‐city time series analysis of 41 cities in 22 countries from March 2nd to 26th 2020. Global Health. 2020;16(1):85. 10.1186/s12992-020-00598-9 32967691 PMC7509494

[43] Douglas M , Katikireddi SV , Taulbut M , McKee M , McCartney G . Mitigating the wider health effects of covid‐19 pandemic response. BMJ. 2020;369:m1557. 10.1136/bmj.m1557 32341002 PMC7184317

[44] Tucker J , Lorig F . Agent‐based social simulations for health crises response: utilising the everyday digital health perspective. Front Public Health. 2023;11:1337151. 10.3389/fpubh.2023.1337151 38298258 PMC10829493

[45] Dignum F , Dignum V , Davidsson P , et al. Analysing the combined health, social and economic impacts of the corovanvirus pandemic using agent‐based social simulation. Minds Mach. 2020;30(2):177‐194. 10.1007/s11023-020-09527-6 PMC7294191 32836870

[46] Song S , Liu X , Li Y , Yu Y . Pandemic policy assessment by artificial intelligence. Sci Rep. 2022;12(1):13843. 10.1038/s41598-022-17892-8 35974068 PMC9379881

[47] Bowser DM , Maurico K , Ruscitti BA , Crown WH . American clusters: using machine learning to understand health and health care disparities in the United States. Health Aff Sch. 2024;2(3):qxae017. 10.1093/haschl/qxae017 38756919 PMC10986293

[48] McCoy D , Mgbara W , Horvitz N , Getz WM , Hubbard A . Ensemble machine learning of factors influencing COVID‐19 across US counties. Sci Rep. 2021;11(1):11777. 10.1038/s41598-021-90827-x 34083563 PMC8175420

[49] Doblhammer G , Reinke C , Kreft D . Social disparities in the first wave of COVID‐19 incidence rates in Germany: a county‐scale explainable machine learning approach. BMJ Open. 2022;12(2):e049852. 10.1136/bmjopen-2021-049852 PMC8852237 35172994

[50] Lim S , Schmälzle R . Artificial intelligence for health message generation: an empirical study using a large language model (LLM) and prompt engineering. Frontiers in Communication. 2023;8:1129082. 10.3389/fcomm.2023.1129082

[51] Karinshak E , Liu SX , Park JS , Hancock JT . Working with AI to persuade: examining a large language model's ability to generate pro‐vaccination messages. Proceedings of the ACM on Human‐Computer Interaction. 2023;7(CSCW1):1‐29. 10.1145/3579592

[52] Wang S , Liang C , Gao Y , et al. Social media insights into spatio‐temporal emotional responses to COVID‐19 crisis. Health Place. 2024;85:103174. 10.1016/j.healthplace.2024.103174 38241850

[53] Lu J , Zhang H , Xiao Y , Wang Y . An environmental uncertainty perception framework for misinformation detection and spread prediction in the COVID‐19 pandemic: artificial intelligence approach. Jmir ai. 2024;3:e47240. 10.2196/47240 38875583 PMC11041461

[54] Barker LA , Moore JD , Cook HA . Generative artificial intelligence as a tool for teaching communication in nutrition and dietetics education‐A novel education innovation. Nutrients. 2024;16(7):914. 10.3390/nu16070914 38612948 PMC11013049

[55] Seagate. Rethink Data . Report reveals that 68% of data available to businesses goes unleveraged. 2020. Accessed August 15, 2024. https://www.seagate.com/gb/en/news/news‐archive/seagates‐rethink‐data‐report‐reveals‐that‐68‐percent‐of‐data‐available‐to‐businesses‐goes‐unleveraged‐pr‐master/

[56] Telenti A , Auli M , Hie BL , Maher C , Saria S , Ioannidis JPA . Large language models for science and medicine. Eur J Clin Invest. 2024;54(6):e14183. 10.1111/eci.14183 38381530

[57] Lv C , Guo W , Yin X , et al. Innovative applications of artificial intelligence during the COVID‐19 pandemic. Infectious Medicine. 2024;3(1):100095. 10.1016/j.imj.2024.100095 38586543 PMC10998276

[58] Yang YT , Wong D , Zhong X , et al. Exploring prior antibiotic exposure characteristics for COVID‐19 hospital admission patients: OpenSAFELY. Antibiotics (Basel). 2024;13(6):566. 10.3390/antibiotics13060566 38927232 PMC11201135

[59] Sarkar C , Das B , Rawat VS , et al. Artificial intelligence and machine learning technology driven modern drug discovery and development. Int J Mol Sci. 2023;24(3):2026. 10.3390/ijms24032026 36768346 PMC9916967

[60] Bali A , Bali N . Role of artificial intelligence in fast‐track drug discovery and vaccine development for COVID‐19. In: Novel AI and Data Science Advancements for Sustainability in the Era of COVID‐19. Elsevier; 2022:201‐229.

[61] Hasselgren C , Oprea TI . Artificial intelligence for drug discovery: are we there yet? Annu Rev Pharmacol Toxicol. 2024;64(1):527‐550. 10.1146/annurev-pharmtox-040323-040828 37738505

[62] Khan MK , Raza M , Shahbaz M , et al. The recent advances in the approach of artificial intelligence (AI) towards drug discovery. Front Chem. 2024;12. 10.3389/fchem.2024.1408740 PMC11176507 38882215

[63] Ge Y , Tian T , Huang S , et al. An integrative drug repositioning framework discovered a potential therapeutic agent targeting COVID‐19. Signal Transduct Targeted Ther. 2021;6(1):165. 10.1038/s41392-021-00568-6 PMC8065335 33895786

[64] van Smeden M , Heinze G , Van Calster B , et al. Critical appraisal of artificial intelligence‐based prediction models for cardiovascular disease. Eur Heart J. 2022;43(31):2921‐2930. 10.1093/eurheartj/ehac238 35639667 PMC9443991

[65] Collins GS , Moons KGM , Dhiman P , et al. TRIPOD+AI statement: updated guidance for reporting clinical prediction models that use regression or machine learning methods. BMJ. 2024;385:e078378. 10.1136/bmj-2023-078378 38626948 PMC11019967

[66] Pilkington E , Aratani L . US transportation, police and hospital systems stricken by global CrowdStrike IT outage. 2024. Accessed July 26, 2024. https://www.theguardian.com/technology/article/2024/jul/19/crowdstrike‐microsoft‐outage

[67] McKee M , Wouters OJ . The challenges of regulating artificial intelligence in healthcare comment on “clinical decision support and new regulatory frameworks for medical devices: are we ready for it? ‐ a viewpoint paper”. Int J Health Policy Manag. 2023;12. 7261. 10.34172/ijhpm.2022.7261 PMC10125205 36243948

[68] Rosenbacke R , Melhus Å , McKee M , Stuckler D . AI and XAI second opinion: the danger of false confirmation in human–AI collaboration. J Med Ethics. 2024. 110074. 10.1136/jme-2024-110074 39074956

[69] Kahneman D . Thinking, Fast and Slow. Farrar, Straus and Giroux; 2017.

[70] Nickerson RS . Confirmation bias: a ubiquitous phenomenon in many guises. Rev Gen Psychol. 1998;2(2):175‐220. 10.1037/1089-2680.2.2.175

[71] Pit P , Ma X , Conway M , et al. Whose side are you on? Investigating the political stance of Large Language models. arXiv preprint arXiv:240313840 2024.

[72] Ke YH , Yang R , Lie SA , et al. Enhancing diagnostic accuracy through multi‐agent conversations: using large language models to mitigate cognitive bias. arXiv preprint arXiv:240114589 2024.

[73] Schmidgall S , Harris C , Essien I , et al. Addressing cognitive bias in medical language models. arXiv preprint arXiv:240208113 2024.

[74] Marcus G . AI platforms like ChatGPT are easy to use but also potentially dangerous. 2022. Accessed August 15, 2024. https://www.scientificamerican.com/article/ai‐platforms‐like‐chatgpt‐are‐easy‐to‐use‐but‐also‐potentially‐dangerous/

[75] Angelov PP , Soares EA , Jiang R , Arnold NI , Atkinson PM . Explainable artificial intelligence: an analytical review. Wiley Interdisciplinary Reviews: Data Min Knowl Discov. 2021;11(5):e1424. 10.1002/widm.1424

[76] García‐Altés A , McKee M , Siciliani L , et al. Understanding public procurement within the health sector: a priority in a post‐COVID‐19 world. Health Econ Pol Law. 2023;18(2):172‐185. 10.1017/s1744133122000184 35894208

[77] Shumailov I , Shumaylov Z , Zhao Y , Papernot N , Anderson R , Gal Y . AI models collapse when trained on recursively generated data. Nature. 2024;631(8022):755‐759. 10.1038/s41586-024-07566-y 39048682 PMC11269175

[78] World Health Oragnisation . Ethics and governance of artificial intelligence for health. 2021. Accessed October 18, 2024. https://www.who.int/publications/i/item/9789240029200

